# Fluvoxamine for the Early Treatment of COVID-19: A Meta-analysis of Randomized Clinical Trials

**DOI:** 10.4269/ajtmh.21-1310

**Published:** 2022-03-09

**Authors:** Christina M. Guo, Ofir Harari, Cameron Chernecki, Kristian Thorlund, Jamie I. Forrest

**Affiliations:** ^1^ Department of Public Health, University of Aberdeen, Aberdeen, Scotland;; ^2^ Cytel Inc., Vancouver, British Columbia, Canada;; ^3^ Department of Health Research Methods, Evidence, and Impact, McMaster University, Hamilton, Ontario, Canada;; ^4^ School of Population and Public Health, Faculty of Medicine, University of British Columbia, Vancouver, British Columbia, Canada

## Abstract

Fluvoxamine is widely prescribed as an antidepressant. Recent studies show the drug may have a clinical benefit in treating COVID-19. We aimed to perform a meta-analysis of the existing randomized trials of fluvoxamine compared with placebo on the early treatment of COVID-19 patients. We included only randomized clinical trials enrolling ambulatory patients with early-stage disease (symptoms < 7 days) for the prevention of hospitalization. We searched MEDLINE and clinicaltrials.gov databases to identify trials and extract data with clarifications from the study investigators. We performed a fixed-effects meta-analysis and sensitivity analyses via R to evaluate the pooled estimate of hospitalization. We included three randomized trials: STOP COVID 1 and 2, and the TOGETHER Trial. The studies included a total of 2,196 patients. The STOP COVID trials measured clinical deterioration whereas the TOGETHER Trial measured hospitalization as the primary outcome. All trials reported on hospitalization up to day 28. The meta-analysis results show that patients receiving fluvoxamine were 31% less likely to experience clinical deterioration or hospitalization compared with placebo (risk ratio, 0.69; 95% CI, 0.54–0.88). A sensitivity analysis using the definition of hospitalization resulted in a risk reduction of 21% (95% CI, 0.60–1.03). Data from three randomized controlled trials show that fluvoxamine was associated with a reduction in the primary outcome measure (either clinical deterioration or composite outcome of hospitalization or extended emergency setting observation), although analysis of hospitalization-only was not statistically significant. More evidence from future trials is still needed to support the findings of this meta-analysis.

## INTRODUCTION

Fluvoxamine is a widely prescribed antidepressant from the selective serotonin reuptake inhibitor (SSRI) class that has been used by clinicians to treat depression, obsessive–compulsive disorder, anxiety, and panic attacks since the mid-1980s (and since the early 1990s in the United States).^1^ The drug has also been shown to exhibit activity on the sigma-1 receptor in the cell endoplasmic reticulum that functions as a modulator of calcium signaling pathways.^2^ In 2019, findings from a mouse model study suggested that agonists of the sigma-1 receptor, such as fluvoxamine, may be clinically useful as treatment of systemic inflammation and sepsis.^3^ Other proposed mechanisms of action for fluvoxamine include reduction in platelet aggregation, decreased mast cell degranulation, interference with endolysosomal viral trafficking, regulation of inositol-requiring enzyme 1α-driven inflammation, and increased melatonin levels, which collectively may exhibit direct antiviral effects.^4^ This pre-clinical evidence has led to a hypothesis that fluvoxamine may have an important clinical benefit for the treatment of COVID-19.

However, despite this scientific rationale for investigating fluvoxamine as a treatment of COVID-19, only a limited number of clinical trials have reported findings. The objective of this study is to conduct a meta-analysis of the existing trials to examine consistency, direction of effect, and where gaps in the knowledge base exist.

## MATERIALS AND METHODS

To be included in our study, we identified randomized trials that included patients of any age receiving fluvoxamine at any dose compared with placebo or any other comparator. Studies had to enroll patients who were classified as early treatment, defined as less than 7 days of symptomology and with a confirmed COVID-19 diagnosis. We excluded studies that were non-randomized.

We searched MEDLINE via PubMed, using the keywords “fluvoxamine” AND “COVID-19,” from January 1, 2019 to December 31, 2021. There were no language or other restrictions applied. We also searched the clinicaltrials.gov database to identify registered trials evaluating the effect of fluvoxamine versus placebo, among COVID-19 patient populations. We contacted and communicated successfully with the principal investigators of identified trials for clarifications and confirmation of our study extraction. We recorded study characteristics, patient populations, interventions, and reported outcomes from included studies, and documented them in Microsoft Excel. The meta-analysis was conducted with *rmeta* in R (version 4.0.5; R Corp Team, Vienna, Austria).

Our primary outcome measure of the effect of fluvoxamine versus placebo for each included trial was clinical deterioration and hospitalization as defined in the study reports. We performed a fixed-effect meta-analysis with risk ratio as the effect measure and corresponding 95% CIs. We assessed statistical heterogeneity using the Cochran’s Q and the I^2^ values. We had predetermined that ad hoc subgroup or sensitivity analyses would be explored if Cochran’s Q yielded a statistically significant signal of lack of homogeneity or I^2^ exceeded 50%. We conducted a sensitivity analysis using the definition of hospitalization, which is in line with modern antivirals in COVID-19. The certainty of evidence for both the study-defined primary outcome and hospitalization was assessed using the Grading of Recommendations, Assessment, Development and Evaluations (GRADE) methodology.

## RESULTS

**Figure 1.**
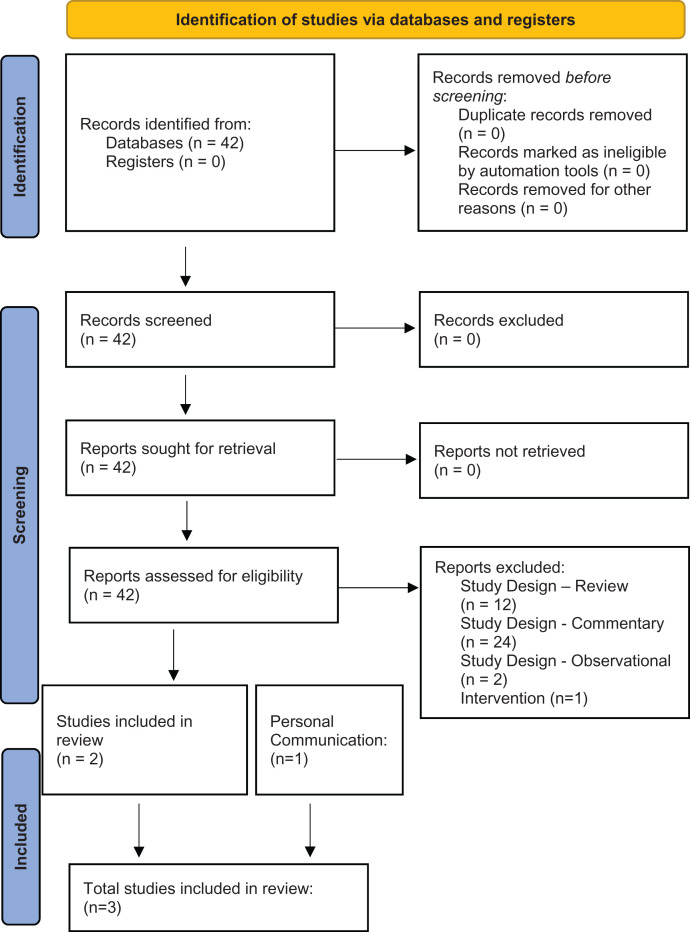
Prisma flow diagram of included studies. *Source:* Page MJ et al., 2021. The PRISMA 2020 statement: an updated guideline for reporting systematic reviews. *BMJ 372*: n71. This figure appears in color at www.ajtmh.org.

A total of 42 studies were scanned (Figure 1) and three distinct randomized trials (STOP COVID 1, STOP COVID 2, and the TOGETHER Trial) were included in this study (Table 1).^5^^,^^6^ Outcomes for both study-defined primary outcome are listed in Table 2, whereas outcomes for hospitalization are listed in Table 3. A search of the clincaltrials.gov database identified an additional three ongoing studies—the Accelerating COVID-19 Therapeutic Interventions and Vaccines (ACTIV-6) sponsored by the National Institutes of Health, the Outpatient Treatment for SARS-CoV-2 Infection (COVID-OUT) Trial sponsored by the University of Minnesota, and the Randomized-controlled Trial of the Effectiveness of the COVID-19 Early Treatment in Community, sponsored by the Chulalongkorn University in Thailand—that are actively recruiting patients, but have not yet reported findings. The University of Minnesota trial and the Chulalongkorn University trial are both scheduled to finish in early 2022, whereas the ACTIV-6 is scheduled to complete in late 2022.

**Table 1:** Description of randomized control trials

Study	Location	Dosing schedule	Inclusion criteria	Exclusion criteria	Total sample size, *n*	Primary outcome measure
STOP COVID 1	United States	50 mg on day 1, 100 mg twice daily on days 2 and 3, 100 mg three times daily on days 4–15	Unvaccinated patients ≥ 18 years Confirmed COVID-19 Symptomatic within 7 days of first dose	Having COVID-19 and requiring hospitalization or showing signs of primary outcome (clinical deterioration) Severe underlying disease Immunocompromised	152	Clinical deterioration within 15 days: shortness of breath/pneumonia and oxygen saturation < 92% or supplemental oxygen required
STOP COVID 2	United States and Canada	50 mg on day 1, 100 mg twice daily on days 2–15	Unvaccinated patients ≥ 30 years Confirmed COVID-19 test and symptomatic within 7 days of first dose One risk factor required	Having COVID-19 and requiring hospitalization or showing signs of primary outcome (clinical deterioration) Unstable medical comorbidities Taking SSRI or drugs affected by fluvoxamine	547	Clinical deterioration within 15 days: shortness of breath/pneumonia and oxygen saturation < 92%, or supplemental oxygen required
TOGETHER Trial	Brazil	100 mg twice a day for 10 days	Unvaccinated patients ≥ 18 years Confirmed COVID-19 test and symptomatic within 7 days of first dose Risk factor required	Patients treated in primary care or requiring hospitalization Patients who received vaccination Patients using SSRIs	1,497	Composite outcome within 28 days: retention in an COVID-19 emergency setting > 6 hours, or transfer to a tertiary hospital, because of COVID-19

SSRI = selective serotonin reuptake inhibitor.

**Table 2:** Study-defined primary outcomes of trials reporting fluvoxamine vs. placebo (primary outcome)

Trial	Sample size, *n*	Study-defined primary outcome, *n* (%)	Risk ratio	95% CI
STOP COVID 1				
Fluvoxamine	80	0 (0)	0.07	0.01–1.21
Placebo	72	6 (5.6)	–	–
STOP COVID 2				
Fluvoxamine	272	13 (4.8)	0.88	0.42–1.81
Placebo	275	15 (5.5)	–	–
TOGETHER Trial				
Fluvoxamine	741	79 (10.7)	0.68	0.52–0.88
Placebo	756	119 (15.7)	–	–

**Table 3:** Hospitalization outcomes in trials reporting fluvoxamine vs. placebo

Trial	Sample size, *n*	Hospitalization, *n* (%)	Risk ratio	95% CI
STOP COVID 1				
Fluvoxamine	80	0 (0)	0.10	0.01–1.83
Placebo	72	4 (5.6)	–	–
STOP COVID 2				
Fluvoxamine	272	9 (3.3)	0.91	0.38–2.20
Placebo	275	10 (3.6)	–	–
TOGETHER Trial				
Fluvoxamine	741	75 (10)	0.79	0.59–1.05
Placebo	756	97 (13)	–	–

STOP COVID 1, a randomized clinical trial conducted by the University of Missouri, was the first to evaluate the effect of fluvoxamine versus placebo.^5^ In total, 152 patients with a confirmed diagnosis of COVID-19, symptom onset in the previous 7 days, and an oxygen saturation more than 92% were assigned randomly to receive either 100 mg fluvoxamine (*n* = 80) or placebo (*n* = 72) three times daily for 15 days. The primary outcome was clinical deterioration within 15 days of randomization defined by meeting both criteria of 1) shortness of breath or hospitalization for shortness of breath or pneumonia and 2) oxygen saturation less than 92% on room air or the need for supplemental oxygen to achieve an oxygen saturation of 92% or greater. Clinical deterioration was reported in zero patients in the fluvoxamine arm, whereas 6 cases were reported in the placebo arm (absolute risk difference, 8.7%; 95% CI, 1.8–16.4). Four of the cases of clinical deterioration in the placebo group required hospitalization (risk ratio [RR], 0.10; 95% CI, 0.01–1.83).^5^

STOP COVID 2 was a follow-up study also measuring differences in clinical deterioration between patients randomized to receive either 100 mg fluvoxamine or placebo for 15 days. The trial was stopped for operational futility, citing increasing vaccination rates in the patient population leading to an overall decrease in the number of observed events. At discontinuation, 13 of 272 patients experienced clinical deterioration in the fluvoxamine group compared with 15 events of 275 in the control group (RR, 0.88; 95% CI, 0.42–1.81). Nine cases of hospitalization were reported in the fluvoxamine group compared with 10 in the placebo group (RR, 0.91; 95% CI, 0.38–2.20).

The TOGETHER Trial^7^ is the largest trial to date to report findings of an evaluation of fluvoxamine versus placebo for the treatment of COVID-19.^6^ In total, 1,497 patients recruited from 11 study sites in Minas Gerais, Brazil, were randomized to receive 100 mg fluvoxamine twice daily for 10 days (*n* = 741) or placebo (*n* = 756).^6^ The primary outcome was hospitalization, defined as retention in a COVID-19 emergency setting for more than 6 hours or transfer to a tertiary hospital as a result of COVID-19 up to 28 days post-randomization. The TOGETHER Trial reported 79 of 741 primary outcome events in the fluvoxamine group and 119 of 756 primary outcome events in the placebo group (RR, 0.68; 95% CI, 0.52–0.88).^6^ Hospitalization occurred in 75 patients in the fluvoxamine group and in 119 patients in the placebo group (RR, 0.79; 95% CI, 0.59–1.05). Death occurred in 17 of 741 patients in the fluvoxamine group and in 25 of 756 patients in the placebo group in the intention-to-treat population.

**Figure 2.**
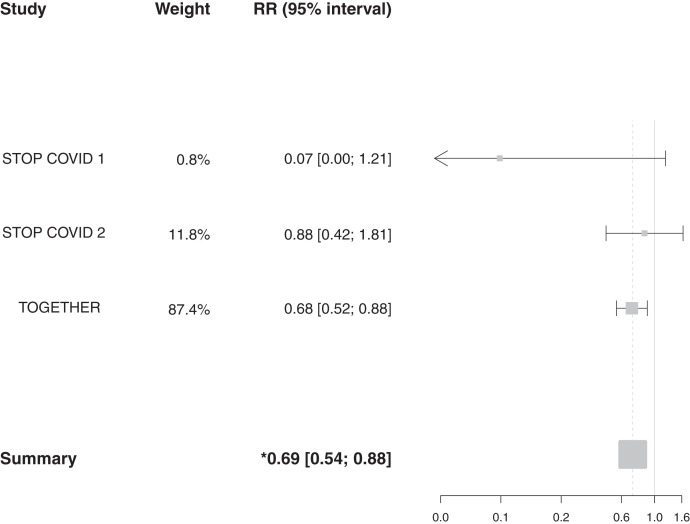
Meta-analysis of studies reporting an effect on study-defined primary outcome of fluvoxamine vs. placebo among adult outpatients with an early diagnosis of COVID-19. RR = risk ratio.

**Figure 3.**
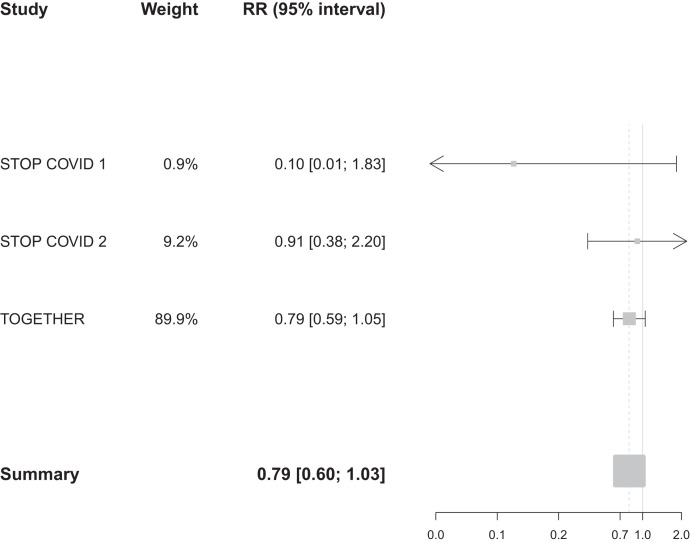
Meta-analysis of studies reporting an effect on hospitalization of fluvoxamine vs. placebo among adult outpatients with an early diagnosis of COVID-19. RR = risk ratio.

Results of our meta-analysis of the effect of fluvoxamine versus placebo on clinical deterioration in the STOP COVID trials and the effect of fluvoxamine versus placebo on hospitalization or extended observation in a COVID-19 emergency setting in the TOGETHER Trial are shown in Figure 2. Patients receiving fluvoxamine, compared with placebo, in these trials were 31% less likely to experience the study-defined primary outcome (RR, 0.69; 95% CI, 0.54–0.88; I^2^ = 31.4%; Cochrane’s Q, 2.92). The majority of evidence for this meta-analysis was weighted to the TOGETHER Trial (weight, 87.4%). In a sensitivity analysis using hospitalization data only, the RR was 0.79 (95% CI, 0.60–1.03; I^2^ = 1.9%; Cochrane’s Q, 2.04) (Figure 3). The risk of bias was considered low and all studies were masked. A GRADE analysis showed that the certainty of evidence for the study-defined primary outcome is moderate, because outcomes include clinical deterioration and hospitalization and are not directly comparable, whereas the certainty of evidence for hospitalization was high (Supplemental Figure S1).

## DISCUSSION

This meta-analysis of reported findings from three randomized clinical trials of fluvoxamine for the treatment of patients with COVID-19 found a significant clinical benefit. The current evidence supports the use of fluvoxamine for the early treatment of COVID-19. Further clinical trials of fluvoxamine will provide further inferences on whether this cheap, generic drug is an important tool for COVID-19.

There are some small differences among the trials. Although the STOP COVID trials allocated patients to receive either 100 mg fluvoxamine three times daily or placebo equivalent for 15 days, the TOGETHER Trial allocated participants to receive either 100 mg fluvoxamine twice daily for 10 days or placebo. Both trials were blinded and used an inert placebo pill as a control. However, although all patients randomized to the placebo arm of the STOP COVID trials took pills three times daily for 15 days, patients allocated to the placebo arm of the TOGETHER Trial received a varying dosing schedule, proportionate to the dosing schedules of the concurrent active treatments in the platform trial at that time. Further trials are required to determine the optimal dosing schedule for COVID-19, because the STOP COVID trials used a 15-day treatment schedule whereas the TOGETHER Trial used a 10-day treatment.

STOP COVID 1 and 2 evaluated the effect of fluvoxamine versus placebo on clinical deterioration of patients within 15 days post-randomization, whereas the TOGETHER Trial evaluated the effect of fluvoxamine versus placebo on hospitalization within 28 days of randomization. Despite these differences, both studies assessed a similar outcome to measure the clinical worsening of patients resulting from COVID-19 and are therefore appropriate for inclusion in this meta-analysis. The adoption of a clinical trial end point for hospitalization, similar to that measured in the TOGETHER Trial, has become increasingly common among COVID treatment trials. However, although hospitalization is a useful measure in COVID-19 studies, hospitalization decisions may differ depending on geographic location and the resource constraints on the system at a given time, which may affect the interpretability of the data collected. Clinical deterioration is arguably a better measurement given changes in the pathology of COVID-19 and may improve the generalizability of the data. In addition, the three trials excluded vaccinated patients, who tend to have a lower rate of hospitalization, which may affect the generalizability of results. Therefore, the absolute effect size is likely less in vaccinated patients than reported by these studies.

In addition, a retrospective cohort study analyzed a database of 83,584 patients diagnosed with COVID-19 in the United States, and matched patients based on demographics, comorbidities, and medication indication to compare patients who were treated with SSRIs to patients not treated with SSRIs. That study found that the relative risk of mortality was reduced in patients prescribed fluoxetine (46 of 470, 9.8%) compared with those untreated (937 of 7,050, 13.3%; RR, 0.72; 95% CI, 0.54–0.97), and those taking either fluoxetine or fluvoxamine (48 of 481, 10.0%) compared with the control (956 of 7,215, 13.3%; RR, 0.74; 95% CI, 0.55–0.99), whereas those receiving an SSRI that was not fluoxetine or fluvoxamine did not have a significant risk reduction. This further suggests that fluoxetine and fluvoxamine should be trialed further to prove an effective treatment of COVID-19.^8^^,^^9^

Limitations of our analysis are predominantly linked to the small number of possible included randomized controlled trials, and the search was limited to one database and one clinical trials registry. In addition, the different primary outcomes of clinical deterioration and hospitalization used in these trials make the meta-analysis more difficult to interpret. Given the small number of included randomized controlled trials, it is impossible to detect heterogeneity, subgroup effects, or the presence of methodological effects. Strategies to detect publication bias were mitigated by contact with experts in the field and searches of registered clinical trials. It remains possible, however, that studies exist that we were unable to identify using standard systematic review approaches.

Identifying safe and effective therapeutics for early treatment in community settings is critical to reducing the morbidity and mortality caused by COVID-19,^10^ particularly for low- and middle-income countries where COVID-19 vaccine access is lowest.^11^ A number of novel therapeutic candidates—notably, those developed by Merck (Merck, Kenilworth, NJ; molnupiravir)^12^ and Pfizer (Pfizer, New York, NY; PF-07321332; ritonavir)^13^—have released promising findings, but it is uncertain whether these drugs will be universally accessible to low- and middle-income countries in the near future. In contrast, a repurposed drug such as fluvoxamine, at an average cost of USD4 per patient, is an immediate treatment solution to an urgent global health crisis.

Although fluvoxamine appears to work via anti-inflammatory pathways, it seems obvious that with the new antiviral drugs, this will result in combination strategies. Some groups are already evaluating fluvoxamine in combination with inhaled budesonide; others are examining the class effect with fluoxetine plus budesonide. There is clearly a need for trials now of molnupiravir and PF-07321332 with anti-inflammatory interventions.

## Supplemental Material


Supplemental materials
