# Supplementary material for: Fluvoxamine for the Early Treatment of COVID-19: A Meta-analysis of Randomized Clinical Trials
Source: Am J Trop Med Hyg. 2022 Mar 9;106(5):1315–20. doi: 10.4269/ajtmh.21-1310 (PMC9128689; doi:10.4269/ajtmh.21-1310)
Supplement: Supplementary file 1 [file tpmd211310.SD1.pdf]

Supplemental Figure S1: GRADE level of certainty for fluvoxamine compared to placebo for COVID-19

Question: Fluvoxamine compared to Placebo for COVID-19
Setting: Outpatients

Certainty assessment							№ of patients		Effect		Certainty	Importance
№ of studies	Study design	Risk of bias	Inconsistency	Indirectness	Imprecision	Other considerations	Fluvoxamine	Placebo	Relative (95% CI)	Absolute (95% CI)		
**Primary Outcome**												
3	randomised trials	not serious[a]	Serious[b]	not serious[c]	not serious[d]	none	94/1093 (8.6%)	143/1103 (13.0%)	**RR 0.69** (0.54 to 0.88)	**40 fewer per 1,000** (from 60 fewer to 16 fewer)	⨁⨁⨁◯ Moderate	IMPORTANT
**Hospitalization**												
3	randomised trials	not serious[e]	not serious[f]	not serious[g]	not serious[h]	none	84/1093 (7.7%)	111/1103 (10.1%)	**RR 0.79** (0.60 to 1.03)	**21 fewer per 1,000** (from 40 fewer to 3 more)	⨁⨁⨁⨁ High	IMPORTANT

CI: confidence interval; **RR:** risk ratio

Explanations

a. No issues were detected as studies have a low risk of bias
b. Outcomes are not directly comparable as a combination of clinical deterioration and hospitalization is used
c. No issues were detected with indirectness
d. No issues were detected with imprecision
e. No issues were detected as studies have a low risk of bias
f. No issues were detected as outcomes are directly comparable
g. No issues were detected with indirectness
h. No issues were detected with imprecision
